# From Pulmonary Embolism to Chronic Thromboembolic Pulmonary Hypertension: A Pathophysiological Approach

**DOI:** 10.31083/j.rcm2511402

**Published:** 2024-11-18

**Authors:** Parham Shahidi, Luise Mentzel, Stephan Blazek, Dmitry Sulimov, Holger Thiele, Karl Fengler

**Affiliations:** ^1^Department of Cardiology, Heart Center Leipzig at University of Leipzig, 04289 Leipzig, Germany

**Keywords:** pulmonary embolism, chronic thromboembolic pulmonary hypertension

## Abstract

Venous thromboembolism presenting as deep vein thrombosis or pulmonary embolism (PE) remains to be an important cause of mortality and morbidity worldwide. Despite its significance and incidence, compared to many other cardiovascular conditions there are significant gaps in knowledge in many aspects of it, including its pathophysiology. A rare sequela of PE is chronic thromboembolic pulmonary hypertension (CTEPH). This complication has a poor outcome and data is scarce in this field. Many therapeutic approaches are based solely on clinical expertise, which can be explained by the complex and not fully understood pathobiology of this disease. Over the years, many theories have been proposed regarding its genesis. Although generally acute PE is accepted as a trigger for CTEPH, this condition is multifactorial and cannot be explained by recurring PEs only. By reviewing the current evidence, we have demonstrated that thrombus non-resolution in CTEPH is due to multiple mechanisms and predisposing factors including: inflammation, small-vessel disease, impaired angiogenesis, platelet dysfunction, coagulopathies, malignancy, impaired fibrinolysis, genetics and many other components. Based on the current evidence, we aimed to explain the pathophysiology CTEPH, PE and the connection between these two important diseases. Furthermore, we highlight the negative hemodynamic effects of CTEPH and PE on the right ventricle and its role in further exacerbation of these patients.

## 1. Introduction

Chronic thromboembolic pulmonary hypertension (CTEPH) is a rare and progressive disease with a poor prognosis, currently classified as group 4 in the World Health Organization’s clinical classification of pulmonary hypertension (PH) [1, 2]. The diagnosis of CTEPH is based on clinical findings (typically including fatigue and excretion dyspnoe), detection of thrombotic material in the pulmonary vascular system and eventually confirmation of the findings by showing elevated precapillary pressure via right heart catheterization [3]. The diagnosis of this disease remains a challenge, with a reported 14 month gap between the initial symptoms and the final diagnosis in centers specializing in this field [4]. We believe this has in part to do with our understanding of the pathophysiology of this disease. Currently, Ventilation-Perfusion mismatch scintigraphy in combination with echocardiography is the recommended method of choice for screening CTEPH according to the European Society of Cardiology [2]. In recent years the diagnosis of CTEPH has been more frequent, most probably due to improvements of diagnostic methods (such as dual-energy computer tomography) and our comprehension of CTEPH [2, 3]. An important distinction of CTEPH from other PH groups is in its specific therapeutic options [4]. Pulmonary endarterectomy (PEA) has proven to be an effective option for more proximal lesions [2, 4]. For more distal lesions and microvaculopathy, balloon pulmonary angioplasty (BPA) and medical therapy are recommended respectively [2]. It is generally accepted that a thromboembolic event plays a key role in the pathogenesis of this disease [5]. Thanks to research done in this field, it is evident that this disease has a multifactorial and complex pathogenesis, which cannot be merely explained by recurrent pulmonary embolisms (PE). In this article we review the pathobiology of PE, discuss the possible mechanisms catalyzing and causing CTEPH and the cardiac consequences of CTEPH as a chronic condition.

## 2. PE

Venous thromboembolism (VTE) presenting as deep vein thrombosis (DVT) or clinical apparent PE is the third most frequent cardiovascular event with significant mortality and morbidity worldwide [6]. Due to silent PE, it is impossible to calculate the exact incidence of PE. However, it is estimated to have an annual incidence of 39–115 per 100,000 population (in Germany approximately 98 per 100,000) [7, 8].

### 2.1 Predisposing Factors

According to Virchow’s triad, hypercoagulability, alteration in blood flow causing stasis and endothelial injury are the three main factors responsible for VTE. Many conditions can act as a predisposing factor by changing one or more components of the Virchow’s triad (Table 1, Ref. [9, 10]): Strong risk factors such as major general surgery, polytrauma and spinal cord injury have an odds ratio of over 10 with an indication for prophylactic treatment against VTE [9]. Cancer is a well-known risk factor for PE, depending on the type, stage, age, treatment factors and time from diagnosis [11]. Highest risks are reported in pancreas, brain, lung, ovarian, lymphomas, myeloma, kidney, stomach and bone cancer [12]. Meanwhile breast and prostate cancer are associated with relatively low risks [12].

**Table 1.** **Acquired and inherited risk factors of VTE [9, 10]**.

Risk	Acquired	Inherited
Weak risk factors (odds ratio <2)	Bed rest (>3 days)	Hyperhomocysteinemia
	Extended immobilization (e.g., air travel)	Homozygous factor XIII 34Val alleles
	Increasing age (≥40 years)	
	Laparoscopic surgery (e.g., cholecystectomy)	
	Obesity	
	Pregnancy/antepartum	
	Varicose veins	
Moderate risk factors (odds ratio 2–9)	Central venous lines	Factor V Leiden mutation
	Chemotherapy	Prothrombin 20210A ^1^
	Congestive heart or respiratory failure	Non-O blood group
	Hormone replacement therapy	Fibrinogen gamma 10034T ^2^
	Malignancy	
	Oral contraceptive therapy	
	Paralytic stroke	
	Postpartum	
	Previous VTE	
Strong risk factors (odds ratio >10)	Lower extremity fracture	Antithrombin deficiency
	Hip/knee replacement	Protein C/S deficiency
	Major Trauma	Tissue factor pathway inhibitor (TFPI) insufficiency
	Spinal cord injury	Endothelin protein C receptor (EPCR) insufficiency
	Major surgery	

1: mutation in the 3^′^-untranslated part of the prothrombin gene, 2: C to T variant at position 10034 in the fibrinogen gamma chain. VTE, venous thromboembolism.

Among the hereditary risk factors antithrombin deficiency, protein C and protein S deficiencies, activated protein C (APC) Resistance, homozygous factor V Leiden mutation, homozygous prothrombin *G20210A* mutation, antiphospholipid antibody syndrome, elevated levels of several coagulation factors, including factors VIII, IX, and XI have been shown to be higher in patients with VTE [7, 9, 13, 14].

Inflammation is known to cause a hypercoagulable state [15]. Logically, autoimmune conditions such as systemic lupus erythematosus, inflammatory bowel disease, immune thrombocytopenic purpura, polyarteritis nodosa and Behçet’s syndrome have been linked to VTE [15, 16]. Furthermore, infectious diseases including Human Immunodeficiency Virus (HIV), respiratory tract, genitourinary tract, and hepatobiliary tract can increase the risk of VTE [17, 18]. Estrogen-containing oral contraceptives as well as post-menopausal hormonal therapy increase the risk of VTE, especially combination compounds containing progesterone and estrogen with up to a 6-fold higher risk [19, 20, 21, 22]. These observations could not be confirmed in progesterone only pills or intra-uterine devices [19].

### 2.2 Sources of PE

The most frequent source of PE are the lower extremities’ deep veins [23]. DVT most commonly occurs in calf veins followed by femoropopliteal veins, and less frequently in the iliac veins [24]. In the presence of predisposing factors such as pelvic infection, pelvic surgery and pregnancy, pelvic vein DVTs can also develop [23]. Proximal veins of the lower extremities or calf vein DVTs with central extensions are more likely to cause a PE compared to only distally located thrombosis [23, 25]. Upper limb DVTs are typically associated with central venous catheters, intracardiac devices such as pacemakers and defibrillators comparatively cause PE less frequently, although with similar outcomes [26, 27]. Although considered a rare occurrence, PE can occur in the absence of peripheral thrombosis or *in situ* PE is also possible [28]. This has been associated with chest trauma, congenital anatomical anomalies, tuberculosis, coronavirus SARS-CoV-2 infection (COVID-19) infection and pneumonectomy [29]. The exact frequency is not known, since differentiating a complete DVT dislodgment and *in situ* pulmonary thrombosis is almost impossible [29]. Furthermore, in many cases a complete exclusion of peripheral thrombosis (e.g., upper extremities) is not performed [28].

### 2.3 Hemodynamic and Respiratory Alterations in PE

PE causes both, hemodynamic alterations and gas exchange interference [18]. In patients without prior cardiopulmonary disease, a non-preconditioned right ventricle (RV) can generate up to a mean pulmonary arterial pressure (mPAP) of 40 mmHg through increased RV wall tension, myocyte stretch and the Frank-Starling mechanism in acute PE [18, 23, 30, 31]. Beyond this point, severe PE which is usually defined as >50% obstruction of the pulmonary circulation, can lead to RV failure [23, 31]. Increased RV wall tension leads to prolonged RV myocardial shortening time into left ventricle (LV) diastole and leftward septal bowing of the heart [32]. This desynchrony can be further exacerbated by right bundle branch block [18]. Due to interference of LV-filling, cardiac output can be massively reduced and lead to hemodynamic instability and consequently to cardiogenic shock [33].

A major reason for hypoxia in PE is due to the redistribution of blood to areas of the lung that are less or not ventilated leading to ventilation-perfusion mismatch [23, 31, 34, 35]. This mismatch causes an increase in anatomical and physiological dead space ventilation and interference with CO₂ elimination [23, 31]. In response, the medullary chemoreceptors increase total minute ventilation, thereby normalizing arterial PCO₂ and respiratory alkalosis [23, 31]. Further contributing factors are intrapulmonary and intracardial shunts [23, 31]. Intrapulmonary shunts can be caused by collapsing alveoli due to lack of surfactant, interalveolar hemorrhage or pulmonary infarction [18, 23, 31, 36]. The increasing pressures of the right side of the heart may lead to intracardiac shunting through the foramen ovale (either a persistent one or separation of the membranes of a closed one) [23, 31, 36]. An additional application of positive end expiratory pressure due to mechanical ventilation can result in a further increase of pulmonary vascular resistance (PVR) [31].

Neurohormonal mediators, mainly thromboxane-A3 and serotonin released by platelets, can cause vasoconstriction further increasing the PVR [37]. Endomyocarditis of the RV assumed to be related to high levels of epinephrin, might explain the clinical exacerbation observed in patients 24–48 hours after the acute PE [18, 38]. Although RV infarction is very rare, elevated levels of biomarkers of myocardial injury hint at ischemia of the RV probably due to an imbalance in oxygen supply and systemic hypotension [18, 39, 40].

While some patients fully recover from an acute PE, up to 16% remain with a persisting impairment of their exercise capacity. In very few cases, development of a CTEPH can be observed [41].

## 3. Role of PE in CTEPH

### 3.1 Incidence and Prevalence of CTEPH and PE

CTEPH is a notoriously underdiagnosed and under-researched disease [2, 36]. There is evidence of geographical differences in CTEPH prevalence, the true incidence however remains unknown [5, 42]. Reports of CTEPH after PE vary from 0.1 to 10% [42]. For the USA and Europe most studies suggest 3.5–4.0% of patients with prior VTE develop CTEPH, while around 75% of CTEPH patients in Europe and North America have a history of acute PE and 56% have had a prior DVT [43, 44, 45, 46]. These numbers seem to be lower in Asian countries, only 15–30% of CTEPH patients in Japan have reported a VTE in their history [44, 47, 48]. CTEPH tends to develop in the first 2–3 years after an acute PE incidence [49, 50]. It is important to note that these numbers regarding PE incidence in CTEPH patients are most probably overestimated, since the first manifestation of CTEPH might be misdiagnosed and recorded as PE [51]. It has been observed that CTEPH patients with a history of PE generally have an asymptomatic phase before developing chronic symptoms [52]. On the other hand, in many cases, CTEPH is diagnosed only a few months after the PE indicating that CTEPH was most likely already present at the index PE [51]. Furthermore, in many of the patients the systolic pulmonary artery pressure (sPAP) is already significantly increased, indicating a chronic process and RV remodeling [51]. Nevertheless, according to these data, PE plays an important role in CTEPH and its pathophysiology.

### 3.2 Thromboembolic vs. Arteriopathy Theory

Based on the thromboembolic theory, CTEPH is primarily triggered by an acute or recurrent PE that fails to resolve [53, 54]. Various factors play an important role in stabilizing a dislodged thrombus and the progression of the disease [53, 54]. That being said, the reason why only a small percentage of patients fail to resolve the fresh thrombi and progress into CTEPH remains speculative [51]. It is nearly impossible to cause CTEPH in an animal model even when inducing recurrent PE and considering that <50% CTEPH patients could be diagnosed with DVT [5, 55]. These observations have led to the formulation of an alternative hypothesis that a primary arteriopathy and secondary *in situ* thrombosis is the main pathological element in CTEPH [56]. An important observation supporting this theory is the formation of pulmonary thrombosis in PH patients of other etiologies [57]. However, differentiating a patient with PH and *in situ* thrombosis with a distal type CTEPH patient is certainly not an easy task and there might be some overlapping between the disorders [5]. Nevertheless, considering the current evidence the role of PE cannot be ignored and *in situ* thrombosis is likely a co-factor in CTEPH genesis.

### 3.3 Predisposing Factors for CTEPH

Looking at the epidemiological data, following predisposing factors have been identified to play a role in the non-resolution of thrombus. Larger, more proximal PE with larger perfusion defects and recurrent emboli are associated with a higher risk of causing CTEPH and not being dissolved efficiently by the fibrinolytic system [1, 49]. One study showed that VTE and CTEPH patients more commonly had non-type O blood [58]. Possible explanations include elevated levels of von Willebrand factor, factor VIII, P-selectin and tumor necrosis factor in non-type O blood [58]. Similarly, hypothyroidism may be diagnosed more frequently in CTEPH patients [58]. It is not exactly known whether thyroid dysfunction or the hormone replacement therapy is the underlying cause [58]. Splenectomy increases the risk of CTEPH probably due to abnormal erythrocytes that would normally be filtered, without which may possibly lead to reactive thrombocytosis [59]. VTE history, history of malignancy, idiopathic PE and age >60 years have all been associated with a higher risk of developing CTEPH [49, 58, 60]. The above-mentioned data have been driven from statistical analysis and their exact molecular mechanisms are still unclear. Further factors that also play a role are discussed below.

Interestingly, intermediate-risk PE (European Society of Cardiology (ESC) classification) has been shown to have higher risk of CTEPH compared to high-risk PE [49]. One likely explanation for this finding could be a more aggressive therapeutic approach in high-risk patients and use of thrombolytic treatment [49]. That being said, the use of thrombolytic treatment alone cannot explain the lower incidence of CTEPH in high-risk PE. In fact, in the PEITHO trial thrombolytic treatment failed to reduce dyspnea and RV dysfunction in intermediate to high-risk PE [61]. In this trial, the CTEPH diagnostic algorithm was not part of the protocol and a definitive diagnosis was made in only 4 out of 190 patients. Therefore, no definitive conclusions can be made [61]. Up to this date there have been no prospective randomized trials conducted investigating the role of an aggressive PE treatment for prevention of CTEPH. However, there has been effort made to find the possible predictors of CTEPH after a PE. In a study unprovoked PE (Odds ratio = 20), onset of symptoms >14 days before diagnosis (Odds Ratio = 7.9), RV dysfunction at presentation (Odds Ratio = 4.1) and hypothyroidism (Odds Ratio = 4.3) were shown to correlate with CTEPH development [62]. It is mportant to note that this correlation does not translate into causation and the background of it remains unclear. Interestingly, in this study contradictory to the PEITHO trial results, patients treated with thrombolytic agents or a surgical embolectomy did not show signs of CTEPH in their follow-ups [62]. Looking at the above-mentioned evidence it becomes clear that our understanding of CTEPH is still to be improved and an accurate interpretation of these findings remains challenging. Currently it is unclear if a timely recanalization treatment reduces the risk of CTEPH.

### 3.4 Mechanistic Studies 

There have been many studies on animals, mostly pigs, dogs, rabbits and mice, trying to explain the mechanism of CTPEH [45]. Reproducing CTEPH features in animals, namely a persistent increase in pulmonary pressure, stable reduction of pulmonary vasculature and most importantly RV remodeling has proven to be more of a challenge than anticipated [63, 64]. In these studies, merely repeated embolization of thrombotic material did not result in long-term complications as observed in CTEPH patients, but rather only a mild increase of sPAP [56, 65]. In a study, use of ligation of the left main pulmonary artery resulted in persistent increased pressures in the pulmonary system. This approach, however, seems to be overly mechanistic and does not explain the molecular and pathological background of CTEPH [66]. Similarly in pigs, the use of spring rings with tissue adhesives created a histologic and physiologic condition similar to CTEPH in humans [56]. In many of the animals used for these studies, the fibrinolytic system is much more efficient [63]. For example, in dogs the faster rates of PE lysis were attributed to higher urokinase-type PA (u-PA) activity and closer association of u-PA with platelets and pulmonary endothelial cell [67]. To overcome this, other approaches such as use of non-thrombotic material as embolus or inhibiting fibrinolysis via the use of tranexamic acid were examined [61, 62], which remains controversial [63]. While some studies have claimed to replicate CTEPH with it and even show impaired angiogenesis, other studies could not observe similar effects [63, 68]. Although some studies have been able to create high stable pulmonary pressures using non-thrombotic materials in rats and rabbits, these particles simulate the thromboembolic process in humans very poorly [63, 68]. In light of these animal experiments, no single method has been proven to be optimal for replicating CTEPH in animal models. Although they have provided us with important information, not all of them translate well into human pathophysiology. In conclusion, these observations point to a complex and multifactorial pathophysiology, which cannot be explained only by recurrent embolisms.

## 4. Other Factors in the Pathophysiology of CTEPH

### 4.1 Small Vessel Disease 

It was first noted by Dr. Moser and his colleagues after performing the first PEA that open pulmonary arteries compared to distal arteries of the occlusion had marked structural changes [69]. These findings were later on confirmed in histological samples and therefore a two-compartment pulmonary vascular bed theory was proposed [5]. These changes include intimal thickening, eccentric intimal fibrosis, intimal fibromuscular proliferation and plexiform lesions affecting distal pulmonary arteries and even arterioles and venules [1, 51]. Due to proximal occlusion the same amount of blood volume flows into a reduced vascular bed, exposing these open pulmonary vessels to higher pressure and shear stress, which is probably an important factor for these histological findings [5, 51]. However, microvasculopathy could also be detected not only in open vessels but also in post-occlusion pulmonary vessels [1, 42]. These changes have been attributed to the thromboembolic mediated pressure increase of the pulmonary system leading to the opening of the pre-existing anastomoses between bronchial (systemic circulation) and pulmonary arteries as well as venules distal to the obstruction [70]. These anastomoses are opened by the pressure gradient generated due to thromboembolic occlusion of the pulmonary system [70]. Backing up this speculation is dilatation of bronchial arteries observed in CTEPH patients [71]. These formed anastomoses supply the ischemic tissue with blood and at the same time results in exposure to systemic pressures [51]. Rarely, failure of this anastomosis formation can lead to stasis of blood in the vessels distal to the obstruction and cause formation of thrombosis [51]. In summary, vascular remodeling can be histologically detected not only in open pulmonary vessels but also vessels distal to the occlusion.

Since small-vessel disease is not treatable with PEA or BPA, it may result in persistent sPAP [72, 73]. Moreover, patients with advanced small-vessel disease are at a higher risk of postoperative mortality [74]. Microvasculopathy can be suspected in cases of an unproportionate high sPAP compared to the extent of proximal occlusion [75]. In these patients, medical treatment with drugs like riociguat is the main therapy.

### 4.2 Coagulation and Fibrinolysis Abnormalities

Most common hereditary thrombophilic conditions such as protein C/S deficiency, factor V mutation and antithrombin deficiency, are not more frequent in CTEPH patients compared to the healthy population [76]. Antiphospholipid antibodies, lupus anticoagulants, levels of clotting factor VIII and Von Willebrand factor have been shown to be higher in CTEPH patients [76, 77, 78]. ADAMTS13, responsible for regulating the size of von Willebrand factor, is also decreased in CTEPH patients even after reduction of sPAP by performing PEA [79].

Another important aspect is failure of a thrombus resolution, possibly due to damage of the fibrinolytic system. Here, many potential enzymes and factors play a role. In a study by Vuylsteke *et al*. [80] tissue plasminogen activators (t-PA) and plasminogen activator inhibitor-1 (PAI-1) were significantly higher in CTEPH patients. In contrast, Lang *et al*. [81] could not observe any significant difference in t-PA and PAI levels. Therefore, abnormalities of t-PA and PAI-1 alone cannot explain the incomplete dissolution of thrombi in CTEPH. In another study, thrombin-activatable fibrinolysis inhibitor (TAFI) was significantly higher in CTEPH patients and remained unchanged after BPA, suggesting an important role in reduction of thrombolysis in CTEPH pathophysiology [82].

Dysfibrinogenemia can also cause resistance of fibrinogen to the fibrinolytic pathway. Up to now, many fibrinogen abnormalities have been shown to be higher in CTEPH leading to thrombus stabilization [83, 84]. That being said, these abnormalities are not unique to CTEPH and can be seen in other types of PH [85]. To conclude, although certain coagulopathies are more frequent in CTEPH patients, no single disorder has been shown to be solely responsible for CTEPH.

### 4.3 Platelet Function

The fact that platelet-favoring conditions such as splenectomy and thyroid hormone replacement therapy are predisposing factors for CTEPH, hinting at the role of platelets in its pathophysiology [51]. In CTEPH patients, platelets have decreased count, higher mean volume, increased spontaneous aggregation and decreased aggregation in response to agonists, suggesting a state of higher platelet turnover [86]. According to a study, platelets in CTEPH patients express P-selectin, PAC-1 binding and guanosine-5’-triphosphate (GTP)-bound GPase RhoA more compared to the healthy population [87]. These markers are responsible for platelet aggregation. Furthermore, platelets showed higher sensitivity to thrombin activation in the CTEPH group [87]. However, these changes were observed in other types of PH, therefore it remains unclear if platelet activation is secondary to increased pressure in the pulmonary vascular system [45, 87]. Lastly, in surgical specimens from PEA, platelet factor 4 levels were increased [88]. Although the above-mentioned evidence does not clearly explain the role of platelets in the pathophysiology of CTEPH, they do suggest that platelet dysfunction is a factor in its pathology.

### 4.4 Impaired Angiogenesis

Pro- and antiangiogenic factors are generally physiologically balanced in way that no angiogenesis happens [89]. This can change under certain circumstances such as tumor formation and wound healing [89]. In animal studies vascular endothelial growth factor (VEGF) and basic fibroblast growth factor (bFGF), major regulators of angiogenesis, are found in organizing thrombi and apparently play a role in thrombus resolution [90]. To further confirm the role of angiogenesis, in an animal study, endothelial-cell-specific deletion of kinase domain protein receptor resulted in ablation of thrombus vascularization and as a result delayed thrombus dissolution [91]. In PEA specimen factors such as platelet factor 4, collagen type and interferon-γ-induced protein-10 (IP-10), which are angiostatic factors, were particularly elevated [89]. Conditions such as splenectomy may also result in higher levels of angiostatic factors such as platelet microparticles and leukocyte-platelet aggregates [92]. Looking at this evidence, angiogenesis is an important step in the resolution of a thrombus. Misguided or deficient angiogenesis can result in thrombus persistence and progression into CTEPH. According to many studies, impaired angiogenesis plays not only a key role in CTEPH pathobiology, but it is also associated with poor prognosis, adverse outcomes, persistent PH post-PEA and start of medical treatment [1, 93].

### 4.5 Role of Inflammation

It is generally well known that inflammation can cause a procoagulant environment. According to epidemiological data, chronic inflammatory conditions such as systemic sclerosis, systemic lupus erythematosus, inflammatory bowel disease, anticardiolipin antibody syndrome and osteomyelitis have been linked to CTEPH [77, 94]. In one case-control study comparing 187 acute PE patients without signs of CTEPH and 109 patients who did develop CTEPH, it was found that up to 10% of the CTEPH patients had a chronic inflammatory condition (odds ratio 67, 95% CI 7.9–8.8) [77]. Ventriculo-atrial (VA) shunts, infected central intravenous lines/pacemakers and chronic infections of indwelling venous catheters are also known predisposing factors [95, 96]. In a study, 6 out of 7 thrombi of VA-shunt carrier patients who underwent PEA contained staphylococcus aureus DNA with upregulation of transforming growth factor beta (TGF-β) and connective tissue growth factor resulting in fibrotic vascular remodeling after thrombosis [97]. These all indicate that persistent infiltration of bacteria causing an inflammatory response and possible thrombus infection are risk factors for CTEPH development.

It has been shown that in PH and CTEPH patients, C reactive protein levels are significantly higher than in a healthy population, and after treatment, C reactive protein levels tends to decrease [98]. Levels of tumor necrosis factor (TNF)-α, interleukin (IL)-6, IL-8, IP-10, monokine induced by interferon-γ (MIG) and monocyte chemoattractant protein-1 α (MIP1α) were also elevated in CTEPH patients, while in idiopathic pulmonary arterial hypertension (IPAH) patients, only MIG and IP-10 were significantly increased [99, 100]. Increased cytokine (MIP1α, IP-10, MIG, IL-6 and RANTES) levels could also be observed in PEA specimens compared to healthy lung tissue [101]. In immunohistological samples of patients undergoing PEA, macrophages, neutrophils, T-lymphocytes and less abundantly B-lymphocytes could be observed. These findings were not as prominent in open vessels [93]. To summarize, in CTEPH patients there is evidence of an over stimulated inflammatory response.

Platelet endothelial cell adhesion molecule 1 (PECAM-1) is a receptor on platelets, endothelial cells, macrophages, neutrophils, lymphocytes, and bone marrow cells responsible for angiogenesis and leukocyte migration [101]. In patients with higher levels of the soluble form of PECAM-1 (sPECAM-1), generated by proteolytic cleavage, delayed thrombus resolution after DVT were observed [102]. Furthermore, animal models with PECAM-1 deficiency led to larger thrombi, further highlighting the possible role of this receptor in CTEPH genesis [102]. In short, it appears that receptors and pathways such as PECAM-1 regulating the inflammatory response play a key role in thrombus resolution.

### 4.6 Genetic Factors

Besides the above mentioned genetic thrombophilic conditions, studies have discovered further possible genetic predisposition factors. In a study using oligonucleotide microarrays, the gene expression profile of pulmonary artery endothelial cells of CTEPH patients were compared to healthy individuals [103]. More than 1600 genes that were differently upregulated or downregulated were identified. Most significantly *JAK3*, *GNA15*, *MAPK13*, *ARRB2* and *F2R* were altered [103]. Angiotensin-converting enzyme polymorphism was shown to be possibly a prognostic factor in the treatment of CTEPH patients [104]. An insertion polymorphism of fibrinogen alpha gene has also been linked to CTEPH susceptibility [105]. Despite the report by Feng *et al*. [106] regarding bone morphogenetic protein type II receptor (BMPR2) and its role in the pathogenesis of CTEPH, but this could not be shown in other studies [107, 108]. Evidence shows the PAH-causing gene mutations, differentially expressed plasma microRNAs as well as increased tissue factor expression also play a role in the pathophysiology of this disease [109, 110, 111]. Increased levels of endothelin-1, a potent vasoconstrictor, and its receptors in the proximal thrombus of CTEPH patients also hints at the role of this pathway and its importance [112]. In summary, not one single gene but rather a combination of mutations and other risk factors seem to be responsible for the pathogenesis of CTEPH.

### 4.7 Malignancy

It is well known that malignancy causes a hypercoagulable state [45]. VTE is a relatively frequent complication in cancer patients, with a 20–30% VTE incidence rate and a three-fold higher risk of VTE compared to non-cancer patients [12, 113]. As expected, Bonderman *et al*. [60], comparing 433 CTEPH patients with 256 healthy individuals, demonstrated a correlation between malignancy and CTEPH (Odds Ratio 3.76, 95% CI 1.47–10.43). Interestingly, recurrent VTE contributed to higher CTEPH incidence, compared to first PE in cancer patients [113]. CTEPH incidence in cancer patients is dependent on the type of cancer as well as the treatment, however the exact mechanism remains disputed [45, 58]. Some of the more common cancers associated with CTEPH include: breast cancer, gastrointestinal carcinoma, melanoma, prostate cancer and seminoma [60]. Possible contributing factors especially relevant in patients with malignant diseases are either a delay in treatment due to lack of symptoms, indwelling vein catheters or neutrophile reduction in the course of chemotherapeutic treatment, which is important in thrombus resolution [45]. Although, management and severity of CTEPH has not been shown to be different in cancer patients, the survival rate are lower in cancer patients (65%) compared to non-cancer patients (89%) [114]. All this being said, the incidence of CTEPH in patients with active malignancies is low and routine screening of CTEPH does not seem to be feasible [113].

### 4.8 Calcium Homeostasis

**Fig. 1.**
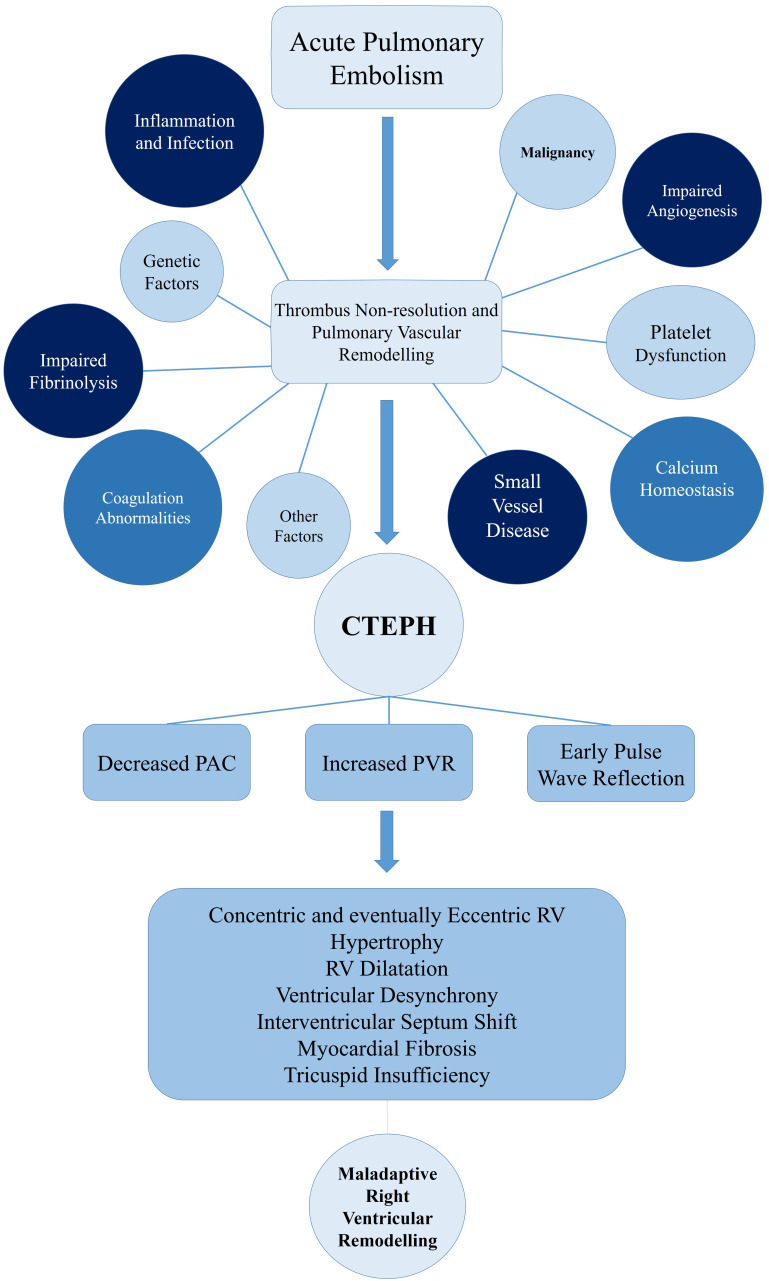
**Pathophysiology of CTEPH und right ventricular remodeling**. CTEPH, chronic thromboembolic pulmonary hypertension; PAC, pulmonary artery compliance; PVR, pulmonary vascular resistance; RV, right ventricle.

Calcium homeostasis seems to be an influencing factor in vascular remodelling [89]. Using PEA samples of CTEPH patients, it was discovered that angiostatic factors can lead to endothelial dysfunction by changing calcium homeostasis [88]. Firth *et al*. [115] also discovered that exposure to fibrin and fibrinogen causes abnormalities of intracellular calcium regulation of pulmonary arterial smooth muscle and endothelial cells, which may be an exacerbating factor for vascular changes of CTEPH patients. A summary of the above-mentioned mechanisms is depicted in Fig. 1.

## 5. RV Remodelling in CTEPH Patients 

Consequences of CTEPH on the RV play a central role in the pathophysiology and prognosis of this disease. An unresolved thrombus in the pulmonary vascular system results in increased PVR and decreased pulmonary artery compliance (PAC) [116, 117]. The increased stiffness of the proximal pulmonary artery results in an early pulse wave reflection increasing RV afterload [118]. Ultimately this leads to an overall increase of pressure on RV due to the summation of backward and forward waves [118]. The magnitude of this reflected pressure is similar in both proximal and distal lesions [119]. However, in proximal lesions this pressure reflection returns to the RV earlier, when the RV is more dilated and under more stress [119]. This is a possible explanation for a poorer RV adaption in these proximal lesions [119]. The increased afterload initially results in RV hypertrophy, which maintains the stroke volume and the exercise capacity of these patients [118]. This initial response is beneficial, hence named the adaptive phase [51, 116, 117]. Eventually, chronic stress leads to exhaustion of the RV adaptation and a so-called maladaptive phase begins [51]. This phase is characterized by eccentric hypertrophy, RV dilatation, reduced RV contractile force and diastolic dysfunction [51]. The increased oxygen consumption of the RV predisposes the tissue to fibrosis, further exacerbating the RV function [116]. Due to ventricular interdependence in the pericardial space, the dilatation of RV leads to septum shift and alterations in LV geometry, reduction of cardiac output and reduction of the coronary perfusion [117]. Similar to the above-mentioned mechanisms of acute PE, the interventricular desynchrony that evolves leads to RV contraction beyond pulmonary valve closure, therefore further increasing RV wall stress [120]. Annular dilatation of the RV and tricuspid insufficiency leads to increased RV preload, further deteriorating this cycle (Fig. 1) [117].

Tsubata *et al*. [121] investigated pulmonary blood flow dynamics in patients with CTEPH and discovered decreased flow velocity and wall shear stress (WSS), an increased oscillatory shear index (an index of the fluctuation of the WSS), and blood stagnation in CTEPH patients. These changes might be at least partially responsible for the hypercoagulability in these vessels [121]. These dynamics were improved with some differences in both BPA and PEA [121].

Molecular mechanisms of this maladaptive response are not fully understood. The TGF-β pathway seems to play an important role in myocardial fibrosis [122, 123]. Additionally, there is a shift of energy metabolism from fatty acid oxidation to glycolysis leading to lactic acid accumulation and hence myocardial damage [124]. RV hypertrophy is linked with disturbed angiogenesis and reduced expression of VEGF [125]. Other possible molecular mechanisms are mitochondrial dysfunction, beta-receptor overactivation, increased endothelin-1, increased cytokine release and increased apoptosis [116]. The above-mentioned changes and maladaptive responses do not seem to be irreversible [117]. There is evidence of a dramatic improvement of RV function after successfully performing PEA in CTEPH patients [126].

## 6. Discussion

By looking at the current literature it becomes clear that there are many disparities in terms of prevalence of CTEPH varying from 0.1 to 10% and also prevalence of prior PE in CTEPH patients [41]. This indicates the lack of our understanding of this complex disease and possibly difficulties that we have in diagnosing it. Despite some competing theories, such as the above-mentioned arteriopathy theory, prior PE is accepted as a central pathophysiological factor by most scientists. Deriving from the statistical analysis that has been completed, one can see many similarities between acute PE and CTEPH, again highlighting a relationship between these two disorders. Although none of the animal-based studies could replicate CTEPH thoroughly, they could show that recurrent PE cannot cause CTEPH by itself [56, 65]. Important to keep in mind is that, most of the data originate from statistical analysis and the molecular background is yet to be discovered.

Another feature of CTEPH, which has been known and histologically proven for many years by Dr. Moser, is the vascular remodelling resulting from changed hemodynamics of the pulmonary vessels [1, 51, 69]. Some of these changes for e.g., decreased flow velocity and WSS are not only responsible for vascular remodelling but also increase coagulability, which could have direct clinical and therapeutic implications [121]. The data regarding coagulopathies and impaired fibrinolysis, are less conclusive and many of the classical coagulopathies do not seem to impact the genesis of CTEPH [76]. However, their role cannot be disregarded and t-PA abnormalities as well as dysfibrinogenemia have been correlated with CTEPH [80, 83]. A similar pattern could also be observed by reviewing the literature for platelet dysfunction, needing more research to define its exact role in the pathophysiology of CTEPH. Angiogenesis and inflammation both seem to play a central role in the resolution of a PE and there has been extensive evidence gathered over the years in support of this [1, 51]. From the current evidence, not a single inflammatory or angiogenetic factor could be recognized as being solely responsible, and direct therapeutic as well as clinical consequences are yet to be understood [98, 99, 100, 101, 102].

Many other mechanisms and risk factors, yet again mainly stemming from statistical correlations, possibly play a role. These include malignancies, genetic factors, hypothyroidism and blood-group type [45, 59, 60]. Another important aspect of CTEPH is its impact on RV-function. By understanding the hemodynamics of RV in CTEPH patients, it becomes more evident that these changes directly play a role in disease progression and possibly further exacerbate the hypercoagulable state [121].

## 7. Conclusions

CTEPH remains an under-researched and largely under-diagnosed condition that is generally associated with a poor outcome. By reviewing the current literature, we could demonstrate that acute PE appears to be the initial trigger of this disease. However, this alone cannot explain the reason behind thrombosis non-resolution in these patients. Other important factors that play an important role include: impaired angiogenesis, small-vessel disease, disbalances in platelet function/coagulation, impaired fibrinolysis, genetics, malignancy and a persistent activated inflammatory cascade. This is an accepted conclusion according to the current published literature. Most of the data leading to these conclusions are based on statistical analysis and the exact underlying molecular mechanisms remain to be discovered. In order to understand the correlation of acute PE and CTEPH better, we also analysed the pathobiology of PE. Similarities in predisposing factors and pathophysiology of these two disorders therefore became clearer. Additionally, consequences of CTEPH on RV function are not only a bystander, but rather directly lead to further progression of this disease. A deeper understanding of this disease could also facilitate the development of more effective therapeutic approaches and improve the prognosis of CTEPH.
